# Virulence Potential and Treatment Options of Multidrug-Resistant (MDR) *Acinetobacter baumannii*

**DOI:** 10.3390/microorganisms9102104

**Published:** 2021-10-06

**Authors:** Sunil Kumar, Razique Anwer, Arezki Azzi

**Affiliations:** 1 Department of Biotechnology, Maharishi Markandeshwar (Deemed to Be University), Mullana, Ambala 133207, India; sunilpgi85@gmail.com; 2 Department of Pathology, College of Medicine, Imam Mohammad Ibn Saud Islamic University (IMSIU), Riyadh 13317-4233, Saudi Arabia; razainuddin@imamu.edu.sa; 3 Department of Biochemistry, College of Medicine, Imam Mohammad Ibn Saud Islamic University (IMSIU), Riyadh 13317-4233, Saudi Arabia

**Keywords:** *Acinetobacter baumannii*, virulence factors, antibiotic therapy, mechanism of action, animal models, Gram-negative bacteria, multidrug resistance

## Abstract

*Acinetobacter baumannii* is an opportunistic pathogen which is undoubtedly known for a high rate of morbidity and mortality in hospital-acquired infections. *A*. *baumannii* causes life-threatening infections, including; ventilator-associated pneumonia (VAP), meningitis, bacteremia, and wound and urinary tract infections (UTI). In 2017, the World Health Organization listed *A. baumannii* as a priority-1 pathogen. The prevalence of *A. baumannii* infections and outbreaks emphasizes the direct need for the use of effective therapeutic agents for treating such infections. Available antimicrobials, such as; carbapenems, tigecycline, and colistins have insufficient effectiveness due to the appearance of multidrug-resistant strains, accentuating the need for alternative and novel therapeutic remedies. To understand and overcome this menace, the knowledge of recent discoveries on the virulence factors of *A. baumannii* is needed. Herein, we summarized the role of various virulence factors, including; outer membrane proteins, efflux pumps, biofilm, penicillin-binding proteins, and siderophores/iron acquisition systems. We reviewed the recent scientific literature on different *A. baumannii* virulence factors and the effective antimicrobial agents for the treatment and management of bacterial infections.

## 1. Introduction

**Figure 1:**
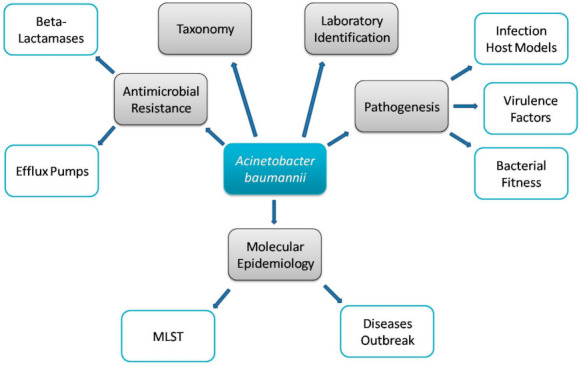
Pathobiology of *A. baumannii*.

*Acinetobacter baumannii* are non-motile, aerobic Gram-negative, glucose non-fermentative, catalase-positive, non-fastidious, oxidative-negative coccobacilli [1]. The phylogenetic classification and interpretation of the taxonomy of the genus Acinetobacterhas established over 60 known species within the genus (Figure 1) [2]. Amongst all other species, *A. baumannii* is particularly associated with hospital-acquired infections (HAI) world wide [3]. Hospital-acquired pneumonia, meningitis, and skin and soft tissue infections are amongst the diverse infections caused by *A. baumannii* [4]. *A. baumannii* has also been known as “Iraqibacter” due to the infections among soldiers admitted to the US military hospitals during the Iraq and Afghanistan conflicts [5]. *A. baumannii* virulence factors have posed multiple challenges and outbreaks in treatment, particularly in critical patients admitted to the intensive care units (ICUs) of healthcare units [3]. 

Infections due to *A. baumannii* are recently emerging particularly in the hospital setting and constitute a challenge for clinicians to tackle their spread and treatment, especially in the ICUs [6]. Furthermore, multidrug resistance has significantly increased in the past two decades, including; resistance to last-resort antibiotics, such as colistin [7,8]. *A. baumannii* infections are usually very difficult to treat; however, the organism has depicted a relatively low virulence potential in immunocompetent hosts [9]. However, recently, animal infection models have reported *A. baumannii* strains with increased pathogenicity [10,11]. This might be due to phenotypic and genetic adaptations that could increase the resistance and virulence potential of *A. baumannii* [12,13].

Physico-chemical functions, including; cell communication, secretion of macromolecules, and surface-regulated attachments, are key factors for bacterial biofilm formation [14]. Several virulence genes enable *A. baumannii* to be a potent bacterial pathogen in animals and humans [15,16]. K1 surface antigen protein 1, acinetobactin transporters, capsular polysaccharides, outer membrane porins, and iron acquisition systems are some of the factors which, along with acquired antibiotics resistance, have promoted *A. baumannii* as a significant nosocomial pathogen [17,18]. *A. baumannii* has been reported to have a range of virulence genes that code for biofilmformation and its adherence on biotic and abiotic surfaces [19]. These virulence genes also facilitate *A. baumannii* in the viable state under different adverse environmental conditions and protect against antimicrobial agents [20,21,22]. Due to the fast development of severe infections and rising antimicrobial resistance of *A. baumannii,* inappropriate empirical therapy is given to the patient, most often with negative consequences [23]. Several studies have examined the effect of antibiotic exposures on the modulation of the virulence properties of resistant pathogens [24,25,26,27]. Augmentation of virulence in *A. baumannii* has been seen by converting a normally low-virulence organism into a highly virulent one in response to variable antibiotic concentrations [28,29]. Several novel treatment options have recently been approved for treating this deadly bug [30,31]. Herein, we review the virulence factors of *A. baumannii* to assess the relationships between antimicrobial resistance, virulence, and the therapeutic options.

## 2. Multidrug Resistance Mechanisms

*A. baumannii* is capable of harboring acquired resistance to a variety of antibiotics used in therapy. Several antimicrobial agents, such aspenicillins, cephalosporins, tetracyclines, aminoglycosides, and quinolones have been tested as ineffective against *A. baumannii* treatment due to the augmentation of resistance determinants. The mechanism behind this might be the genetic alteration causing modification of the membrane fusion proteins (OMPs); overexpression of antimicrobial modifying enzymes; overexpression of efflux transporters; modification of target sites; and the insertion of new resistance determinants. Consequently, carbapenems are the only choice of treatment alternatives due to their enhanced activity and reduced toxicity for *A. baumannii* infections [32]. In the past few decades, *A. baumannii* causedan array of nosocomial infections, which were successfully treated with gentamicin, minocycline, nalidixic acid, ampicillin, or carbenicillin, either as monotherapy or in combinations, but global surveys have shown an increased resistance in the hospital isolates [33,34,35]. Several mechanisms of resistance have been implicated in the augmentation of resistance in *A. baumannii*:

1. Enzymatic mechanisms including; deferent β-lactamases.
2. Non-enzymatic mechanisms involving efflux pumps and membrane permeability.
3. Change in the sequence of penicillin-binding proteins (PBPs).

Among these, the first two are very crucial in imparting resistance to clinical isolates of *A. baumannii* [36].The action of bacterial β-lactamase resulted in the rise of antibiotic resistance to the penicillins, cephalosporins, and carbapenems [37,38]. *A. baumannii* gives rise to β-lactamases, which are coded by various chromosomal genes or plasmids genes.

### 2.1. Enzymatic Mechanisms (Beta-Lactamases)

Different classes of β-lactam antibiotics become hydrolyzed by the enzymes produced by bacterial cells, such as cephalosporins, carbapenems, penicillins, and monobactam, which are neutralized by β-lactamases. Beta-lactamases are classified into four major classes (A, B, C, and D) as per their gene sequence homology. On the basis ofthe inclusion of divalent cations in enzyme activation, these enzymes are classified into metallo- β-lactamases (class-B) and non- metallo-β-lactamases (classes A, C, and D) [39]. Overproduction of beta-lactamases has been associated with the escalated resistance to carbapenems in *A. baumannii* in many investigations.AmpC beta-lactamases come under the Ambler classification of preferred ESBL (extended-spectrum beta-lactamases) [40]. As per recent information on *A. baumannii*, the most significant mechanism is the carbapenemase behind carbapenem resistance, which is the most powerful class of β-lactamases. Divalent cations (zinc) are utilized by metallo-enzymes in addition to water moleculesin order to cleave the β-lactam ring. Carbapenem hydrolyzing class-D beta-lactamases (CHDL) are considered as another promising cause of carbapenem resistance in *A. baumannii*. These enzymes are also known as oxacillinases (OXAs) because of their capacity to hydrolyzeisoxazolyl-penicillin-oxacillin quicker than benzylpenicillin [41,42]. To date, many types of OXAs have been discovered in the *A. baumannii*, such as OXA-23, OXA-51, OXA-40/24, OXA-48, OXA-58, and OXA-143 [32,43].

Class-A (GES and KPC) and class-B carbapenemases (NDM, IMP, SIM, and VIM) have also been screened in *A. baumannii*. Many class-A carbapenemases are crystallized (e.g., SME-1, KPC-2, andNmcA). Most of the class-C β-lactamases show weaker activity in the hydrolysis of carbapenems. OXA-23 is the most prominently found CHDL beta-lactamase, primarily found on a plasmid of the *A. baumannii* strain in Scotland and represented as the first oxacillinase harboring carbapenemase activity [44]. Later on, the blaOXA-23 gene was identified both on plasmids and on chromosomes all over globe. It is exclusively found in the genus Acinetobacter with the exception of a *Proteus mirabilis* isolate from France [45]. OXA-24/40, initially isolated from Spain, were predicted the first time as separate enzymes but re-sequencing confirmed them as identical later on [46].

OXA-58 has also been identified in *A. baumannii*, which shares a 59% protein sequence similarity with OXA-51/69 [47]. OXA-58 is particularly coded by a plasmid gene, which has been circulated extensively all over the world. Initially, OXA-58 was more prevalent in Greece and Italy [48,49,50,51,52] because of frequent outbreaks in intensive care units and pediatric wards [53,54,55]. Nowadays, OXA-58 is found in *A. baumannii* in every corner of the world [56]. OXA-143 is the more recently identified group of CHDLs, which were formerly traced in the clinical isolate of *A. baumannii* in Brazil [57]. CHDLs hydrolyze the carbapenems typically very effectively; therefore, as a substrate, imipenem is given preference over meropenem and hence raises a debate on the contributing role of CHDLs in carbapenem resistance [58,59]. Some of the studies have tried to prove this by implementing either knock-out mutants or by using transformation experiments to see the changes in susceptibility to carbapenems.

### 2.2. Non-Enzymatic Mechanisms (Efflux Pumps)

Efflux pumps are the components of the bacterial cell membrane which excrete toxic substances, metabolic end-products, and even antimicrobials [60]. Overexpression of chromosomally encoded efflux pumps is one of the several mechanisms responsible for multidrug resistance in *A. baumannii* [61]. Efflux pumps have been presented as the first step in the augmentation of an MDR phenotype [62]. Most of the efflux pumps are prevalently described in the Gram-negative bacteria, which are comprised of three protein components [63]. The resistance-nodulation-division (RND) efflux family, which most commonly fetches antimicrobial resistance in *A. baumannii*, is composed of a periplasmic MFP (membrane fusion protein) interacting with an inner cytoplasmic membrane transporter protein at one end and an OMP (outer membrane protein) on other end to facilitate the extrusion of antimicrobials [64,65]. Such efflux systems are the MexAB-OprM system (previously studied as MexAB-OprK) of *P. aeruginosa* [66] and the AcrAB system in *E. coli*. In the *Burkholderiacepacia* complex, RND efflux pumps have also been explored for the association of antimicrobial drug resistance to different antimicrobials [67]. Apart from the MexAB-OprM system, three more RND efflux systems have been characterized: MexEF-OprN, MexXY-OprM, and MexCD-OprJ [60,68]. To date, many RND efflux pump systems have been described in *A. baumannii*, including; AdeABC, AdeFGH, and AdeIJK [36,69,70,71]. Each of these efflux pumps is regulated by a two-component regulatory system, e.g.,AdeRS regulates the AdeABC in *A. baumannii* [72];AdeL (LysR-type transcriptional regulator) regulates the AdeFGH [70]; and AdeN regulates the AdeIJK for TetR transcriptional regulation [73]. Overexpression of the efflux pumps by up-regulation has led to increased antimicrobial resistance in *A. baumannii* [36,74]. The MF (Major Facilitator) and MATE (Multidrug And Toxic compound Extrusion) super family efflux pumps have also been observed contributing to antimicrobial resistance in *A. baumannii* [69,72,75].

## 3. Virulence Factors

**Figure 2:**
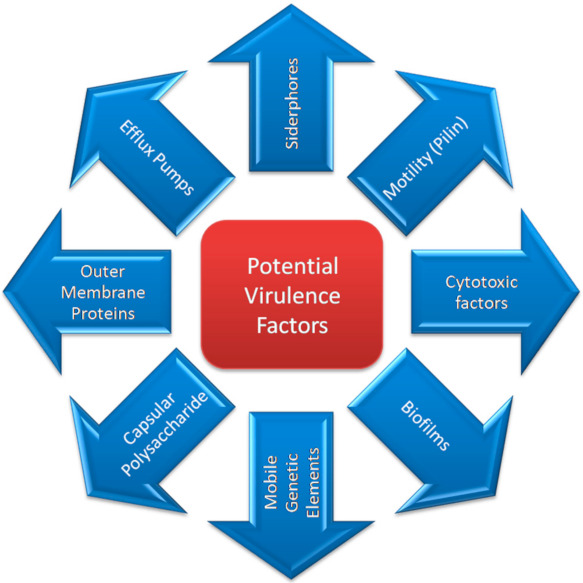
Potential virulence factors of *A. baumannii*.

Multiple virulence factors facilitate *A. baumannii* in passing on a disease to the host efficiently (Figure 2) [15]. Only a meager understanding is available about the *A. baumannii* virulence potential and the host responses to infection caused by this bacterium [76,77]. Virulence factor identification and the underlying mechanisms of pathogenesis can help in the synthesis of new treatment substitutes to control the disease by this bug [24].

### 3.1. Outer Membrane Proteins (OMPs) and Outer Membrane Vesicles (OMVs)

Outer membrane proteins of Gram-negative bacteria are investigated for their association with antimicrobial resistance, pathogenesis, and variation in the adherence of the host cell [78]. *A. baumannii* isolates have shown a few OMPs, which are symbolized as the most prevalent ones among other species as well [79]. *A. baumannii* OmpA (AbOmpA) is reported as an impending virulence factor bringing numerous significant properties to signal processing and pathogenesis [80]. AbOmpA of *A. baumannii* is a major surface-bound protein, which facilitates the attachment process and host cell invasion and helps in the commencement of apoptosis at the onset of infection. OmpA adds to the initiation of cell apoptosis, the invasion of epithelial cells, and serum resistance [81,82]. Gram-negative bacteria generally produce outer membrane vesicles (OMVs) that assist in confronting virulence factors by host cells [83]. Lipo-polysaccharides (LPS), lipids, proteins, and DNA or RNA are the main constituents of OMVs, which have a diameter range of approximately 20–200 nm [84,85]. OMVs deliver the virulence factor to host cells and transfer resistance genes from one bacteria to another [86].

### 3.2. Biofilm

Biofilm is a complex structure of diversified bacterial cells usually found adhered to the biotic or abiotic surfaces, embedded in an extracellular matrix of polymers (involving nucleic acids, carbohydrates, proteins, and other constituents) and representing a shielding mechanism to thrive in unfavorable environmental conditions and at the time of host infection [87,88]. One of the essential virulence factors required for adherence to biotic and abiotic surfaces and biofilm formation is 38 kDa (OmpA) outer membrane protein A [80,89]. Initial attachment to the abiotic surface is mediated by pili production controlled by the usher-chaperone assembly structure (CsuA/BABCDE) and regulated by BfmS/BfmR (a 2-component system) [90]. Formation and regulation of biofilms depend upon several host factors, such as growth condition, cell density, quorum sensing, light, and free iron [91,92]. A significant component of exopolysaccharide is PNAG (Poly-β-1,6-N-acetyl -glucosamine), which constitutes biofilms [93]. The effortless adherence to epithelial cells, diverse medical devices, and equipment is crucial for *A. baumannii* invasion in the susceptible hosts and persistence in hospitals [94]. Such bacteria show a higher resistance against variable antibiotics, stressors, or disinfectants than the motile ones and hence the capacity to produce biofilms symbolizes a critical factor of virulence. Biofilm formation can be manipulated by general features, such as bacterial appendages, bacterial surface constituents, availability of nutrition medium, macromolecular secretions, and quorum sensing systems [91]. The capability of clinical strains of *A. baumannii* to synthesize biofilm is attributed to a resistance phenotype, particularly on abiotic surfaces, catheter-associated infections, sepsis or urinary tract catheters and even in the patients with shunt-related meningitis [95,96,97].

### 3.3. Penicillin-Binding Proteins (PBPs)

PBPs are enzymes usually found as membrane-bound in the cytoplasm of bacteria and share a familiar evolutionary origin [98]. These enzymes are essential for synthesizing peptidoglycan, the bacterial cell wall’s principal constituent, and coupled with cell division and morphogenesis [99]. Disorientation in the cell wall is caused by the inhibition of PBPs, which further inhibit growth or cell lysis. PBPs have been categorized into low and highmolecular weight PBPs. Low molecular weight PBPs help cell segregation and the remodeling of peptidoglycan, whereas highmolecular weight PBPs are responsible for cell wall and peptidoglycan synthesis [100]. Using rat soft-tissue infection models, Russo et al. also showed that a mutant derivative of the wild *A. baumannii* (PBP-7/8 deficient) is added tothe *A. baumannii* pathogenesis and is involved in its survival and growth in the ascites of humans [101]. A few studies also portrayed the role of penicillin-binding proteins in pathogenesis due to *A. baumannii* [102].

### 3.4. Siderophores/Iron

Pathogenic bacteria face one major challenge in their hosts, which is the shortage of freely accessible iron. Iron is almost unavailable in mammals for invading bacteria and is mostly integrated into storage proteins and iron transport. The ability of the host to withhold iron significantly diminishes the accessibility of iron to infecting bacteria and is accounted for as an essential module of innate immunity [17]. Siderophores are secreted and bind to ferric ions and enable *A. baumannii* to capture iron under an iron-shortage condition [103]. Bacterial cells acquire siderophores loaded with Fe^3+^ and heme through specific protein receptors. These receptors are usually present on the outer membrane of Gram-negative bacteria where siderophore-Fe^2+^ complexes are internalized, coupled with the indulgence of the proton gradient on the bacterial inner membrane [104]. TonB protein complexmediates the energy transduction in the periplasmic space from the inner to the outer membrane [105]. Recently, a novel siderophore, cefiderocol (siderophore-cephalosporin conjugate), investigated in a subject suffering from kidney impairment, was found to be well tolerated as a treatment for multidrug-resistant strains [106]. Synthesis of fimsbactin A and B has also been studied recently, based on the synthesis routes as stereoisomeric analogues [107]. A gamma-lactam antimicrobial has been effectively tested upon MDR *A. baumannii* in the USA recently [108]. Some studies also explained the roles of phospholipase D and capsular polysaccharide as virulence factors which add to the pathogenicity of *A. baumannii* [109,110].

## 4. Antimicrobial Therapy

### 4.1. Monotherapy

**Figure 3:**
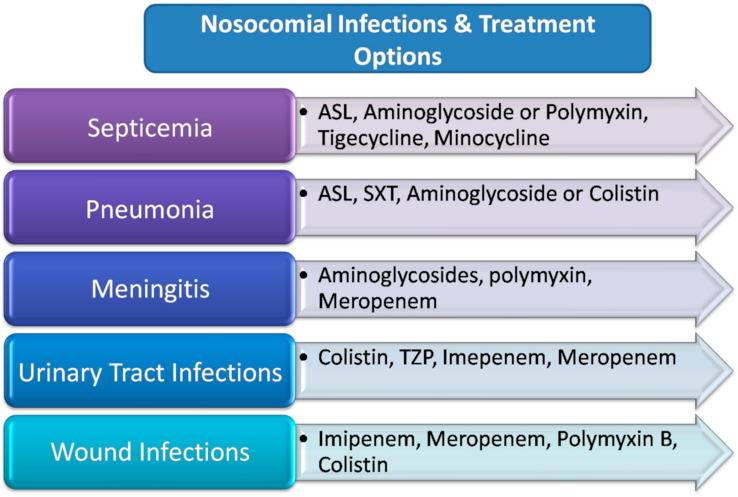
Therapeutic treatment options for nosocomial infections of *A. baumannii*.

*A. baumannii* infections are commonly severe and complicated to cure because of escalating multidrug resistance, specifically among clinical isolates [111,112]. Consecutive surveys have revealed the mounting resistance in clinical isolates. The majority of *A. baumannii* strains have become resistant against clinically attainable doses of nearly all prevalent utilized antimicrobials, such as ureidopenicillins, aminoglycosides, aminopenicillins, fluoroquinolones, chloramphenicol, and cephalosporins [113,114]. The rate of resistance of sulbactam is also rising periodically despite being bactericidal to many other species of the genus Acinetobacter [115]. Carbapenems, in particular meropenem and imipenem, were previously very successful in in vitro treatment. Carbapenems are considered as the last resort of the treatment for managing infections caused by MDR *A. baumannii*. Still, the rate of resistance against carbapenems has increased in clinical strains of *A. baumannii*, particularly in Europe, Latin America, Asia, and Australia (Figure 3) [56,116,117,118]. Such carbapenem-resistant *A. baumannii* (CRAB) isolates are generally resistant to other conservatively used common antibiotics [56,113,119]. Imipenem has a higher affinity for certain oxacillinase enzymes than meropenem. The susceptibility to tigecycline or polymyxins at present remains satisfactory [120,121]. A global surveillance from 2007–2011 suggested the escalating susceptibility of minocycline ranging from 72.5% to 91.7% [122,123]. In a study from Italy, an increase in resistance to the latest generation of antimicrobials has been observed in sepsis cases [124]. Most of the *A. baumannii* isolates have been reported resistant to most of the antibiotics (95–99%) in another investigation [125]. Minocycline showed bactericidal effects against *A. baumannii* isolates as well as synergistic effects with other antimicrobials.

### 4.2. Synergy and Combination Therapy

Although the microbiological clearance rates in the *A. baumannii* severe infections are considerably higher with the use of combined therapy, recent analysis stated that there is an inadequacy of clinical effectiveness (mortality or cure rate) using combination therapy comparative to monotherapy. Absence of regulated clinical trials renders it complicated to judge the effect of combination therapy or synergy for treating MDR *A. baumannii* infections [126]. Combined therapy is recommended when all conventional drugs become ineffective against the *A. baumannii* [127]. Synergy and combinations of drugs through in vitro andin vivoexperiments have shown extremely bactericidal activity against the hospital strains of MDR *A. baumannii* [128,129]. Two or three classes of the following antibiotics are included in such synergic combinations: rifampin, polymixins, sulbactam, tigecycline, aminoglycosides, or β-lactams, such as broad-spectrum cephalosporins or carbapenems [130,131,132]. However, all antibiotics should be tested individually against each strain and combined with suitable in vitro methods as multiple diverse resistance mechanisms are prevalent in the clinical isolates.

Conflicting results have been found in a few studies when different investigators used the same antimicrobial combinations. Montero et al., performed a study on a murine pneumonia host model of MDR *A. baumannii*. They concluded that rifampin combinedtherapy with tobramycin, imipenem, or colistin were the most efficient treatment options [133]. However, a follow-up clinical study suggested not using a combination of rifampin and imipenem for CRAB treatment because of a high failure rate. It acknowledged the increased resistance to rifampin in 70% of treated patients with this regimen [134]. To treat the imipenem-resistant pneumonia using a guinea pig model, amikacin and imipenem combined therapy gave a worse outcome than imipenem alone; however, the in vitro results represented the synergism among the antimicrobials [135]. The in vitro synergy benefits are still not clear. A broad category of additional antimicrobials usage put a limit on the capability to describe the overall outcome of combined therapy. In contrast to colistin parenteral monotherapy, most combination therapy outcomes were equivalent with respect to cure rates. Additional clinical studies with controlled parameters are required to conclude whether any suitable combination of antimicrobials could be translated to useful therapeutic strategies.

### 4.3. Dose and Drug of Choice

The dosage and drug of choice relied upon in vitro susceptibility surveys but not on rigorous clinical trials. The persistence and spread of specific epidemic lineages of *A. baumannii* in the different geographical locations indicatethat information on the prevailing local susceptibility pattern is critical for treating Acinetobacter infection. *A. baumannii* susceptible isolates could be quickly treated with conservative antimicrobial agents, spanning over the third and fourth generations of cephalosporin, fluoroquinolones, or carbapenems [136]. Aminoglycosides in combination with other antibiotics are usually prescribed for treating meningitis or bacteremia and have shown promising activity against *A. baumannii* in in vivo and in vitro studies [119,137]. Tetracycline is also preferred to treat *A. baumannii* infections in several experimental and clinical studies [138].

It is essential to understand the fact that *A. baumannii* clinical isolates are increasingly reported as multidrug resistant and a few of them are resistant to almost all the routinely used antibiotics [139]. Therefore, it is necessary to have complete antimicrobial susceptibility testing [140]. Carbapenems have been the drug of choice for the last 20 years for treating *A. baumannii* infections when susceptibility data were generally not available [141]. However, the outbreak of epidemic clonal lineages worldwide with increased resistance to carbapenems has been documented during the last decade [56,142,143]. Before the availability of antimicrobial susceptibility tests, a combination of carbapenem withother antibiotics (sulbactam, tigecycline, or polymyxins) is perhaps an accepted choice for empirical therapy to treat *A. baumannii* infections [126]. The following antibiotics, either as monotherapy or in combined therapy, have been utilized for treating MDR isolates of *A. baumannii* with a lower success rate.

### 4.4. Polymyxins

While inadequate therapeutic options are in hand, clinicians have revisited the use of polymyxin E (colistin) or polymyxin B for MDR Acinetobacter infections [144]. The mode of action of colistin is the disrupting of the cell membrane by interacting with the lipopolysaccharide, increasing the membrane permeability, and resulting in bacterial cell fatality [145]. Colistin acts as a bactericidal drug and its effect is dependent upon the concentration utilized against *A. baumannii* [146]. There are two routes of colistin medicationwhere colistin sulphate is given through an oral route andfor topical useand, colistin sulphomethate sodium is administered intravenously [147]. Polymyxins, either in mono- or combined therapy, have proved effective intravenously in the clinical outcomes of patients suffering with meningitis or pneumonia (VAP) [148,149]. Toxicity rates, predominantly nephrotoxicity due to colistin, are usually lower than those reported earlier, but some investigators have shown the rates of AKI (Acute Kidney Injury) reaching up to 50% [150]. Lack of alternate antibiotics, failure to examine the renal functions, and various factors for renal injury make it difficult to access the outcomes of such studies. Polymyxins share similar PK-PD (pharmacokinetics/pharmacodynamics) with aminoglycosides; a trim level prospective study demonstrated the safety and efficiency of an elevated dose of colistin in critical patients [151]. Polymyxins in conjugation with other antimicrobial agents in aerosol form can also be given intravenously, which showed effectiveness in patients with *A. baumannii* nosocomial pneumonia [152]. When the intravenous colistin, or other antimicrobials, combined with aerosolized colistin, the outcome was improved in the subjective reports [153]. However, the combination of intravenous colistin had no discriminating benefit in a randomized study [152]. However, in critical patients, the extensive use of polymyxins for treating *A. baumannii* infections may lead to an escalating and increasing resistance [154]. Hetero-resistance to colistin has also been reported in *A. baumannii* isolates [155]. Consequently, combination therapy has been recommended with polymyxins and other antimicrobials [144].

### 4.5. Sulbactam/β-Lactamase Inhibitors

Sulbactam, which is a predominant β-lactamase inhibitor, has an inherent mechanism against *A. baumannii* isolates [30]. In vivo and in vitro assays of sulbactam have shown promising results against *A. baumannii* infections [129]. Ampicillin, a β-lactam agent, combined with the beta-lactamase inhibitor, did not materialize to enhance the synergy or activity [156]. Use of sulbactam alone is not recommended for severe Acinetobacter infections. However, Qin et al. reported extensive sulbactam use in the treatment of 114 patients with MDR Acinetobacter pulmonary infections, resulting in similar clinical outcomes among patients treated with sulbactam and cefoperazone alone [157]. Recently, sulbactam combined with meropenem therapy resulted in decreased biomass and mean thickness, raised the roughness of the Acinetobacter biofilm, and showed synergism against biofilm-embedded CRAB [158]. In another investigation, antimicrobial susceptibility results of MIC tests (broth microdilution method) in 176 isolates of *A. baumannii* of β-lactam/β-lactamase combinations showed a 99.4% susceptibility, which suggested future prophylactic and therapeutic options [159].

According to the particular geographical area, the in vitro susceptibility of *A. baumannii* differs extensively for sulbactam [160]. No substantial randomized clinical trial has been available with sulbactam. Studies have shown that compared to other successful antibiotics for treating resistant *A. baumannii*, sulbactam, either in combination or alone, has shown similar efficacy [161]. A higher sulbactam dose of >6 g/day is recommended in serious patients, and a regimen of 9 g/day of sulbactam has been used effectively, exclusive of significant side effects [162]. However, due to rampant clinical use, the antimicrobial efficacy of sulbactam has declined extensively against *A. baumannii* isolates and resistance to sulbactam is frequently increasing in certain geographical regions [163].

### 4.6. Tigecycline

Tigecycline is a relatively new glycylcycline agent, a synthetic derivative of minocycline, and possesses a specific mechanism of action. Tigecycline has been found to have bacteriostatic activity against CRAB isolates [164]. Some MDR Acinetobacter isolates have reported increased resistance to tigecycline by rapidly evading tigecycline by up-regulating efflux pumps encoded by chromosomal genes [165,166]. Clinical reports described the combination regimens of tigecycline to treat *A. baumannii* infections, such as bacteremia, VAP, soft tissue, and skin infections [126,167]. A poor correlation has been reported through studies between microbiological and clinical outcomes, mainly among patients under treatment for respiratory tract infections [120,168,169]. A low rate (48%) of microbiological eradication with tigecycline treatment has been noticed in *A. baumannii* hospital-acquired pneumonia (HAP) in some patients, probably due to the lack of adequate tigecycline concentration [170]. Another study recommended the high dose of tigecycline over a smalldose by PK-PD interpretation and partial clinical investigations sustain its safety and efficiency [171,172]. In a study of UTI (urinary tract infections) from Italy, 85% of uro-pathogens were Gram-negative bacteria [173]. For treating UTI, tigecycline is not recommended as the right choice drug as it is not excreted via urine [174]. The development of tigecycline resistance in *P. aeruginosa*, *A. baumannii*, and *K. pneumoniae* infections was also reported during therapy [119]. Therefore, it is crucial to avoid tigecycline use as a monotherapy for *A. baumannii* infection treatment. After discussing these findings, tigecycline is considered a good option for salvage therapy of infectious diseases [167,175].

### 4.7. Aminoglycosides

Tobramycin and amikacin are aminoglycoside agents utilized as therapeutic options for infections with MDR *A. baumannii* that keep hold of their susceptibility [176,177]. Such agents are frequently utilized in combination with a different potent antibiotic [178]. Numerous MDR Acinetobacter isolates maintain intermediate susceptibility to tobramycin or amikacin and increasing resistance to these agents is coupled with efflux pump mechanisms of aminoglycoside modifying enzymes [179]. Recently, the role of the outermembrane protein ‘A’ has been associated with increased resistance to gentamicin [180]. However, in vitro studies have reported a rise in resistance to aminoglycosides [181].

### 4.8. Tetracyclines

In a recent study from North India, a combination therapy of tetracycline with nalidixic acid showed a synergistic effect on MDR isolates of *A. baumannii* [182]. Minocycline has shown considerable results through an in vitro pharmacodynamics model against carbapenem-resistant and susceptible isolates of *A. baumannii* in monotherapy and combination with other antimicrobials [123]. Data from a few investigations have shown that more than 90% of isolates of *A. baumannii* were susceptibile to minocycline [183]. Subjective data has recommended minocycline in combinations with other antibiotics through intravenous route in critically ill patients with MDR Acinetobacter infections after the noticeable in vitro activity and constructive pharmacodynamics profiles [184]. However, large scale relative studies with an adequate number of patients are essential to validatethe minocycline efficacy. A study of over 2000 samples was recently conducted with a novel derivative of tetracycline (omadacycline) where 90% of the *A. baumannii* isolates were inhibited with a concentration of ≤4 μg/mL [185]. However, resistance to tetracycline has been reported in a number of investigations due to the overexpression of tet efflux pumps [186].

## 5. Prevention and Control

Due to the high level of antimicrobial resistance, it is very difficult to control the *A. baumannii* infections. It is really difficult to get *A. baumannii* eliminated from a healthcare unit once it gets endemic. However, when a stubborn approach is implemented to infection prevention and control (IPC), it is not impossible to eliminate this organism from a unit. WHO guidelines were enforced in 2017 and should be strictly followed to effectively manage *A. baumannii* infections [187]. Although regular surveillance programs to control the infection are deficient in controlling the transmission of resistant Acinetobacter infections, inclusion of a variety of improvised action plans covering the assurance of all sorts of frontline hospital staff have revealed an indication of improvement (Figure 3) [188]. Detection of the source of transmission, cleaning of the environment, judicious feedback of information and medical equipment disinfection, strengthening of standard precautions, and hand hygiene are all necessary steps to prevent the hospital outbreaks [189]. The patients should be kept in isolated sites; for patients receiving mechanical ventilation, a closed tracheal suction system should be used to prevent contamination [190]. However, there are many other examples where it has been compulsory to put into practice a closure of wards for a period of up to one month, so that outbreaks of *A. baumannii* could be controlled [191] At genome level, further investigations are required to screen novel genomic islands responsible for a high virulence potential [192].

## 6. Study of Virulence Using Animal Models

To study *A. baumannii* pathogenesis and virulence, model systems have now been established, including; in vitro and in vivo systems in invertebrates and mammals [193,194,195]. *C. elegans* has been used as a host model to detect the variations in virulence factors amongst a variety of *A. baumannii* isolates with already identified virulence factors and resistance mechanisms [28]. Several studies have investigated high levels of antibiotic resistance over the last two decades [145]. To a large extent, the in vivo work in association with the quick adjustment of *A. baumannii* in the presence of antibioticshas been conducted by defining its genetic architecture spanning hospitalsettings [195,196]. Several other investigations have also underlined the significance of supplementary virulence factors [197,198]. The knowledge of the association of the fitness cost and the virulence factors with the resistance mechanisms of *A. baumannii* stillis lacking; however, both the virulence and fitness costs are thought to be connected with antimicrobial resistance [196,199]. *A. baumannii* virulence factors and pathogenetic potential allow it to grow and survive in the various habitats and environmental conditions where several other bacteria could not continue to exist [200,201]. Multiple investigations have used mammalian infection models for inventing fitness and virulence assays of *A. baumannii* and other related bacterial pathogens, especially in the rodent models [202]. Vertebrate host models require multifarious infrastructure, amenities and trained manpower to handle, maintain and monitor large number of animals. Therefore, the invertebrate host models have become popular nowadays as alternative host models with less expenditure, a short life span, and low cost maintenance [203,204].

Over the last decade, developed invertebrate models decreased the cost and complications of mammalian model experiments and the need for animal care [205]. Moreover, no ethical issues are involved with the use of invertebrate models [206]. Such merits enable the large-scale infection model trials, which are predominantly utilized for high throughput selection of mutated bacterial strains [207]. Established invertebrate models to study the virulence include *Drosophila melanogaster* [208], *C. elegans* [196], *Galleria mellonella* [203], *Bombyx mori* [209], and *Dictyostelium discoideum* [210].

*G. mellonella* and *C. elegans* have been demonstrated as the leading significant and standard host models in the laboratories to study bacterial virulence and pathogenic islands [211]. As a host model of *A. baumannii*, *C. elegans* possesses numerous merits over *G. mellonella* in targeting the host-bacterial relations, such as a small size; a petite generation time; a transparent body; a short genome; a simple lifecycle; ease of maintenance; no requirement for external suppliers; accessibility of a plentiful choice of genetically modified mutants; the full knowledge of its lineage; and the simplicity of the animal [212,213].

## 7. Conclusions

*A. baumannii* is a notorious, nosocomial pathogen responsible for hospital-acquired infections. It has an ability to survive in a variety of hospital environments and to accumulate antimicrobial resistance. Multidrug resistance is the most troublesome feature of *A. baumannii*, due to which new treatment agents are ineffective against it. Antibiotic resistance mechanisms of *A. baumannii* include β-lactamases; modification of aminoglycosides; overexpression of multidrug efflux pumps; under-expression of outer membrane porins; and modifications of target sites. As per recent studies, polymyxin B, another polymyxin antibiotic, has been proposed as a prospective therapeutic option to colistin. Various animal models are crucial to investigating the pathogenicity and virulenceassociated with *A. baumannii*. *G. mellonella* larvae have identified hundreds of genes necessary for in vivo *A. baumannii* survival. Additionally, other model animals using transposon screening will underscore the probability of a novel insight into the pathogenesis of *A. baumannii*. However, the pathogenicity and toxicity of *A. baumannii* still remain unclear despite recent advancements. Patients with severe infections should be included as hotspots from areas with high resistance rates for targeting future treatment options and trials. Rapid detection and implementation of thorough infection control measures, development of novel antibiotics and maintenance of effectiveness of already available antibiotics are the key factors for controlling the *A. baumannii* infections successfully.

## Data Availability

Not applicable.
